# Unspecific chronic low back pain – a simple functional classification tested in a case series of patients with spinal deformities

**DOI:** 10.1186/1748-7161-4-4

**Published:** 2009-02-17

**Authors:** Hans-Rudolf Weiss, Mario Werkmann

**Affiliations:** 1 Koob-Scolitech, Huehnerhof 100, D-55568 Abtweiler, Germany; 2 Orthomed Orthopedic Technical Services, Bad Sobernheim, Germany

## Abstract

**Background:**

Up to now, chronic low back pain without radicular symptoms is not classified and attributed in international literature as being "unspecific". For specific bracing of this patient group we use simple physical tests to predict the brace type the patient is most likely to benefit from. Based on these physical tests we have developed a simple functional classification of "unspecific" low back pain in patients with spinal deformities.

**Methods:**

Between January 2006 and July 2007 we have tested 130 patients (116 females and 14 males) with spinal deformities (average age 45 years, ranging from 14 years to 69) and chronic unspecific low back pain (pain for > 24 months) along with the indication for brace treatment for chronic unspecific low back pain. Some of the patients had symptoms of spinal claudication (n = 16). The "sagittal realignment test" (SRT) was applied, a lumbar hyperextension test, and the "sagittal delordosation test" (SDT). Additionally 3 female patients with spondylolisthesis were tested, including one female with symptoms of spinal claudication and 2 of these patients were 14 years of age and the other 43yrs old at the time of testing.

**Results:**

117 Patients reported significant pain release in the SRT and 13 in the SDT (>/= 2 steps in the Roland & Morris VRS). 3 Patients had no significant pain release in both of the tests (< 2 steps in the Roland & Morris VRS).

Pain intensity was high (3,29) before performing the physical tests (VRS-scale 0–5) and low (1,37) while performing the physical test for the whole sample of patients. The differences where highly significant in the Wilcoxon test (z = -3,79; p < 0,0001).

In the 16 patients who did not respond to the SRT in the manual investigation we found hypermobility at L5/S1 or a spondylolisthesis at level L5/S1. In the other patients who responded well to the SRT loss of lumbar lordosis was the main issue, a finding which, according to scientific literature, correlates well with low back pain. The 3 patients who did not respond to either test had a fair pain reduction in a generally delordosing brace with an isolated small foam pad inserted at the level of L 2/3, leading to a lordosation at this region.

**Discussion:**

With the exception of 3 patients (2.3%) a clear distribution to one of the two classes has been possible. 117 patients were supplied successfully with a sagittal realignment test-brace (physio-logic^®^ brace) and 13 with a sagittal delordosing brace (spondylogic^®^ brace). There were patients with scoliosies and hyperkyphosiesbrace). Therefore a clear distribution of the patients from this sample to either chronic postural or chronic instability back pain was possible. In 2.3% a combined chronic low back pain from the findings obtained seems reasonable.

**Conclusion:**

Chronic unspecific low back pain is possible to clearly be classified physically. This functional classification is necessary to decide on which specific conservative approach (physical therapy, braces) should be used.

Other factors than spinal deformities contribute to chronic low back pain.

## Background

There is an increasing prevalence of low back pain, spinal stenosis and degenerative scoliosis in the aged population. Even though the exact percentage of patients with a symptomatology of spinal stenosis is not known, the main goal is to provide pain relief and improve functional lifestyle with minimum intervention [1]. There also seems to be an increasing prevalence of spinal stenosis. According to Ciol et al [2] between 1979 and 1992 the incidence of surgery for this condition increased to finally be eight times higher. As to whether sedentary lifestyle contributes to low back pain in adulthood or not is still a matter of discussion. The modern sedentary lifestyle is hypothesized to create disuse changes beginning with muscle, which ultimately causes interference with the adaptive and structural dynamics of specialized connective tissue [3].

A sedentary lifestyle can lead to a loss of lumbar lordosis and this condition correlates with low back pain, spinal claudication, which may further have links with degenerative scoliosis in the adult population [4].

Masiero et al. [5] have shown that non-specific LBP is a frequent event even in teenagers, particularly in females, sedentary children and those with a family history of LBP.

Low back pain has been seen in 1,416 out of 2,346 secondary school pupils (60%), and in 32% of the examined students. Statistical analysis has confirmed a correlation between LBP and such risk factors as the incorrect sedentary position (p < .001 for pupils, and p < .02 for students), and smoking (p < .001 for students and p < .02 for pupils) [6].

Also Hildebrandt and co-workers [7] conclude that stimulation of leisure time physical activity may constitute one of the means of reducing musculoskeletal morbidity in the working population, in particular in sedentary workers.

Sedentary postmenopausal women may benefit from regular long-term therapeutic exercise in terms of subjective back complaints and slowed loss of bone mass.

However there are also studies, which do not support the hypothesis that sedentary lifestyle contributes to low back pain [8-10]. In one study the lordotic angle seemed to have no influence on the prevalence of low back pain [8], but one has to take into consideration, that not the angle of lordosis, but the location of lordosis in the lumbar spine contributes to pain relief or increase [11,12].

The term chronic low back pain relates to patients with pains in the lumbosacral region including pains in the sacroiliac joints. The iliolumbar ligaments can also be involved and radicular symptoms may also be part of this syndrome. When there is a radicular symptom the low back pain may be attributed as being specific, for the nerve root affected determines the origin of the symptom [13].

Chronic low back pain without radicular symptoms cannot be related to one specific nerve root and mostly involves quite a number of symptoms such as pains involving L5/S1 (sometimes also L4/5), the sacroiliac joint and the iliolumbar ligaments [13].

Up to now chronic low back pain without radicular symptoms and without any other specific finding (eg. spondylolisthesis) is not classified and attributed in international literature as being "unspecific". For specific bracing of this group of patients we use simple physical tests to predict the brace type the patient might benefit from. Based on these physical tests we have developed a simple functional classification of "unspecific" low back pain in patients with spinal deformities.

## Methods

Between January 2006 and July 2007 we have tested a total of 133 patients (119 females and 14 males) with spinal deformities (average age 45 years, ranging from 14 years to 69) and chronic unspecific low back pain (pain for > 24 months). Some of the patients had symptoms of spinal claudication (n = 15).

**Figure 1:**
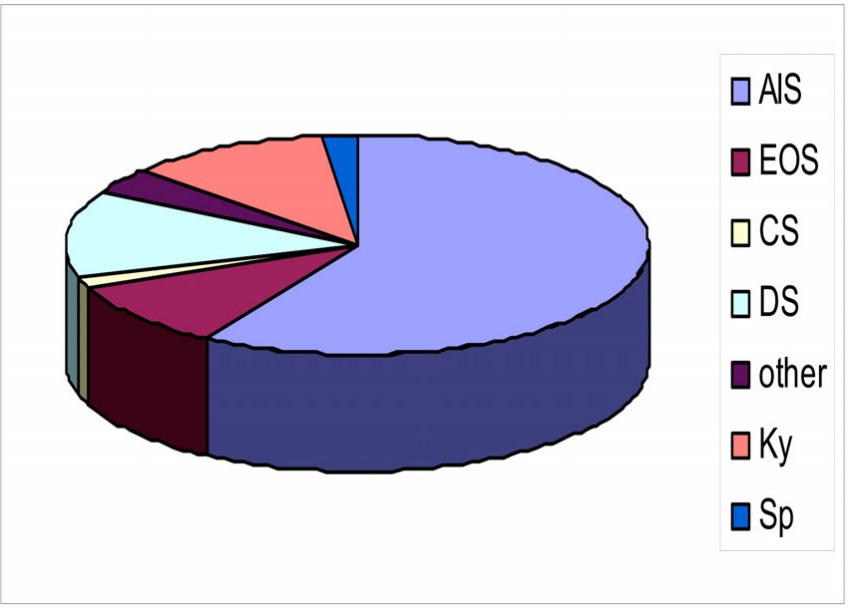
**Distribution of the diagnoses in the sample of patients**. There were patients with scolioses and kyphoses in the sample described. 78 (59%) had an Adolescent Idiopathic Scoliosis (AIS), 13 (10%) had an Early Onset Scoliosis (EOS), 2 (1,5%) had congenital scoliosis (CS), 17 (13%) had degenerative (de novo) scoliosis (DS), 5 (4%) had scoliosis in combination with other deseases (other), 15 (11%) patients had kyposis (Ky) and 3 (2%) had a spondylolisthesis (Sp).

There were patients with scoliosis and hyperkyphosis in the sample described. 78 (59%) had an Adolescent Idiopathic Scoliosis (Cobb angle between 23 and 54°), 13 (10%) had an Early Onset Scoliosis (Cobb angle between 45 and 63°), 2 (1,5%) had congenital scoliosis, 17 (13%) had degenerative (de novo) scoliosis (Cobb angle between 24 and 38°), 5 (4%) had scoliosis of other origin (Cobb angle 34 – 69°), 15 (11%) patients had hyperkyphoses of various degrees and locations (Thoracic, thoracolumbar and lumbar) and 3 (2%) had a spondylolisthesis with additional scoliosis with Cobb angles between 24 and 32° (Figure 1).

No other radiological or other forms of imaging findings have been found than reported here with the exception of age or scoliosis related degeneration, which was not necessarily related to the clinical findings.

There were no obvious demographic differences visible in the groups responding to the different tests with the exception that the patients who had a negative response to both tests belonged to the older ones (45, 63 and 69 years of age).

The indication for brace treatment were as follows:

- no successful conservative treatment (physical therapy, injections, physiotherapy) during the last 24 months and

- the patients wanted to try to prevent surgery.

3 female patients with spondylolisthesis have been tested for brace treatment, one with symptoms of spinal claudication also. Two of these patients where 14 years of age and the other 43.

**Figure 2:**
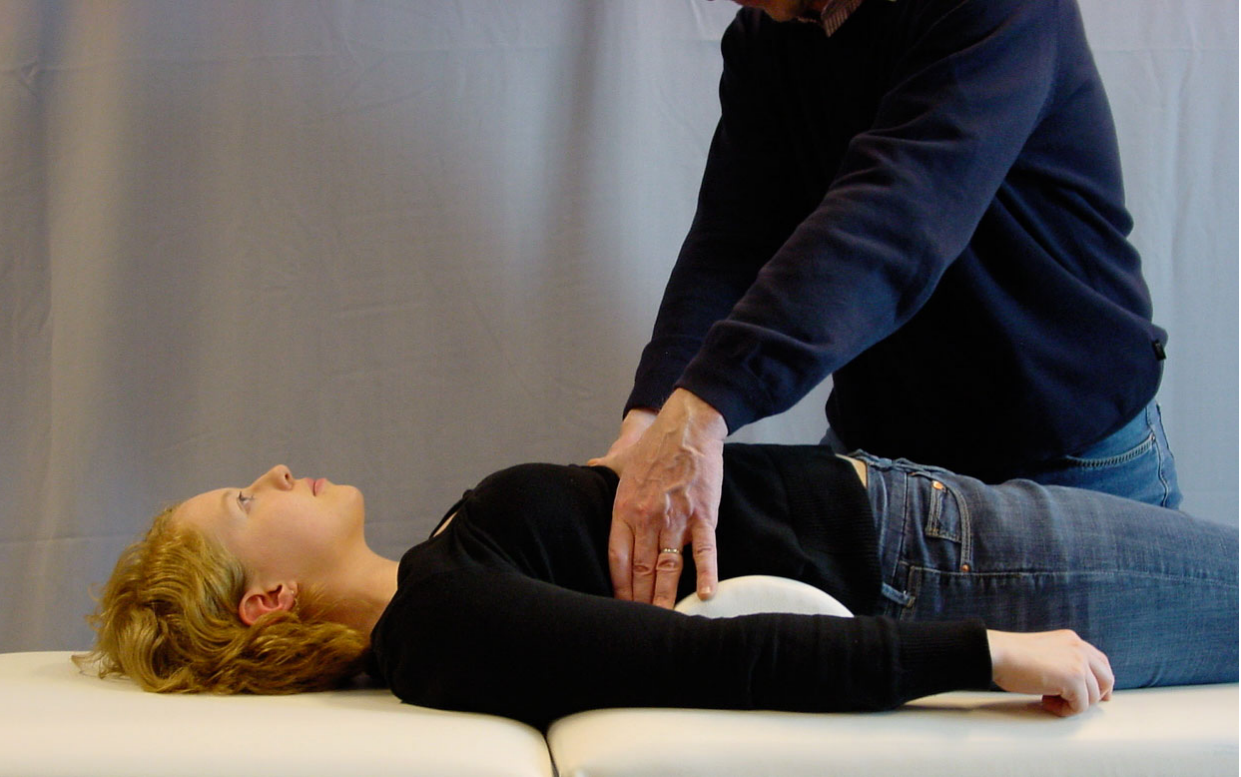
**The Sagittal Realignment Test (SRT)**. Sagittal realignment test (SRT) lying (Weiss 2005) to estimate as to whether a patient will benefit from physio-logic^® ^exercises or the physio-logic^® ^brace. In the positive case this test will immediately reduce chronic LBP (PLBP).

**Figure 3:**
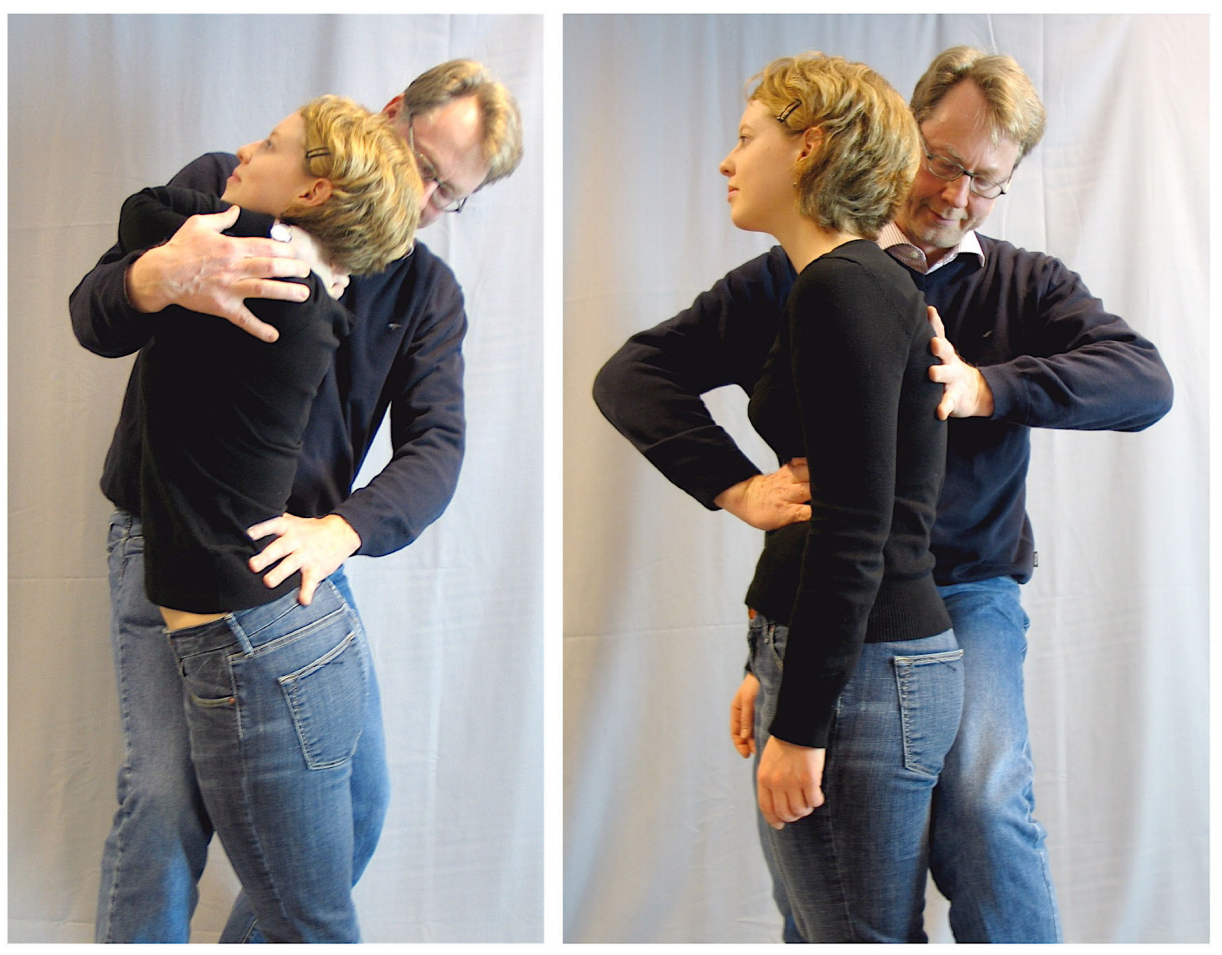
**The Sagittal Realignment Test (SRT) and the Delordosation Test (DT) in standing position**. Sagittal realignment test (SRT) in standing position (left). In the positive case this test will immediately reduce chronic LBP (PLBP), and the delordosation test (DT) (right). In the positive case this test will immediately reduce chronic LBP if this is due to instability (ILBP).

We applied the "sagittal realignment test" (SRT, see figure 2 and 3 on the *left*), a lumbar hyperextension test, and the "sagittal delordosation test" (SDT, see figure 3 on the *right*).

## Results

117 patients reported significant pain release in the SRT and 13 in the SDT (>/= 2 steps in the Roland & Morris VRS). 3 Patients had no significant pain release in both of the tests (< 2 steps in the Roland & Morris VRS). In the 16 patients who did not respond to the SRT in the manual investigation we found a hypermobility L5/S1 in a clinical test or a spondylolisthesis L5/S1 radiographically. In the other patients who responded well to the SRT, loss of lumbar lordosis was the main issue, a finding which, according to actual knowledge, is correlated well with low back pain. The 3 patients who did not respond to either test had a fair pain reduction in a generally delordosating brace with an isolated small foam pad inserted to the level of L 2/3, leading to a lordosation at this specific region.

Pain intensity was high (3,29) before performing the physical tests (VRS-scale 0–5) and low (1,37) while performing the physical test for the whole sample of patients. The differences where highly significant in the Wilcoxon test (z = -3,79; p < 0,0001).

## Discussion

In actual fact scoliosis does not automatically induce spinal or low back pain (LBP) [14] and therefore the type of low back pain as reported in this study should not be restricted to the existence of a spinal deformity. A study with over 2000 scoliosis patients with pain reveals a lack of correlation of pain intensity and curve magnitude [15].

In clinical practice we now distinguish between what we call postural low back pain [PLBP] (related to the loss of lumbar lordosis) and instability low back pain [ILBP] (related to a sagittal instability L4/5 or L5/S1 like in symptomatic spondylolisthesis) in patients with and without spinal deformities. Also a combination of both categories rarely seems possible. Simple physical tests are described to distinguish clinically between the two main categories [16].

**Figure 4:**
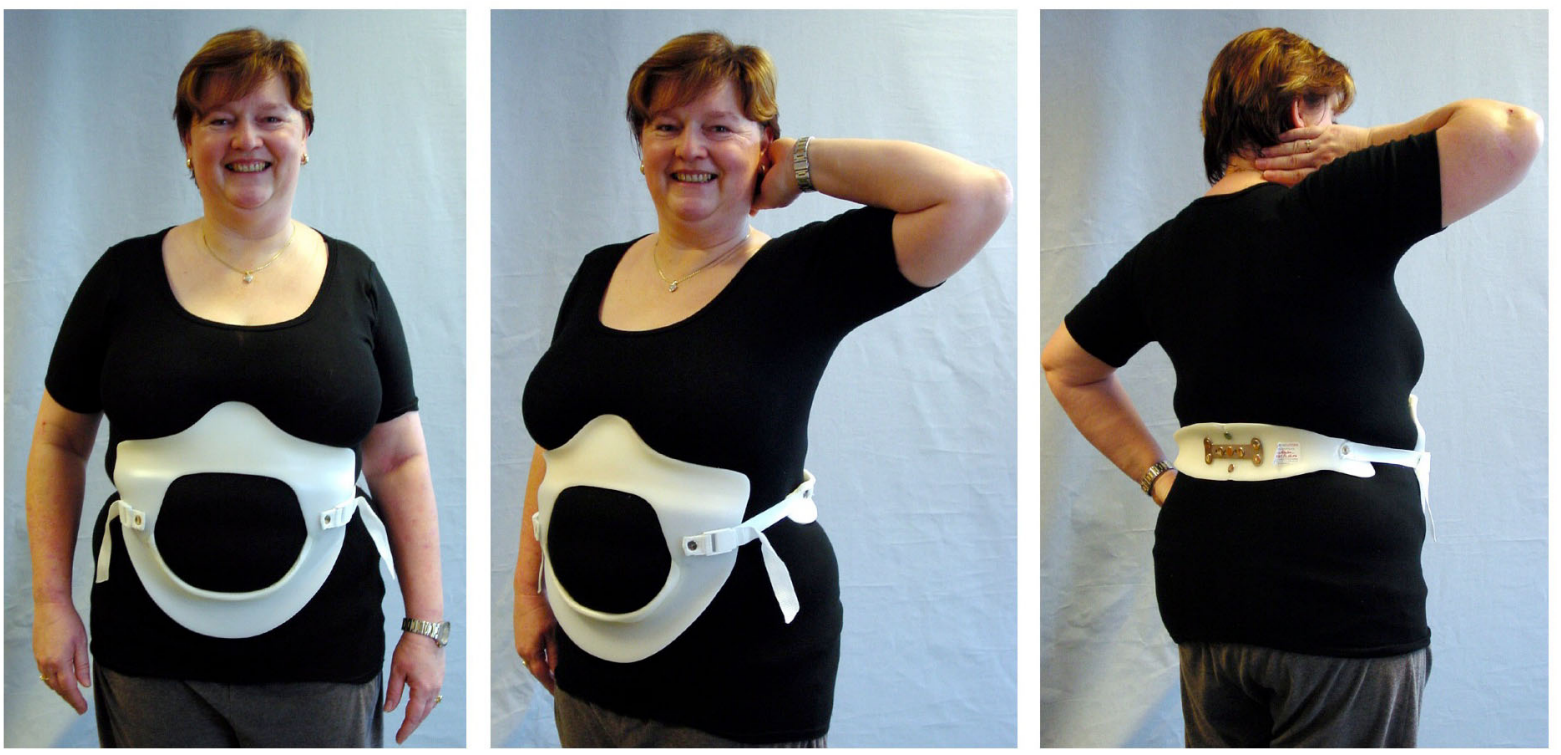
**physio-logic^® ^brace**. physio-logic^® ^brace improving chronic low back pain in this patient with spinal stenosis and increasing walking ability drastically from 500 steps to 12000 steps.

The strategy for physical rehabilitation and bracing differs essentially and can be separated into two basic categories:

Relordosation techniques [17-19] have to be applied in PLBP (about 90% of the LBP population) to improve/correct the sagittal profile aiming at a balanced "S" when viewed from lateral. Bracing strategies should also be specifically aiming at the anatomical sagittal profile [20] and therefore should increase lumbar lordosis with an apex at L2 level [11,12,16]. This bracing strategy is implemented in the physio-logic^® ^brace (Figure 4), which has been shown to decrease intensity of chronic LBP immediately as well as symptoms of spinal claudication, in the majority of these cases [11,12,16].

As it seems the physio-logic^® ^brace uses the internal or "intrinsic" stabilisation system of the lumbar spine by locking the facet joints, preventing lateral movement of the lumbar spine. This stabilisation on the other hand reduces the loads on the ventral column of the spine and by this, releases the pressure the intervertebral discs are exposed to. It has already been demonstrated that lumbar relordosation even corrects a lateral spinal deviation [21], which supports the stabilisation theory.

As a matter of fact, as pointed out by Burwell [22], the function of the lumbar segment is best in lordotic posture and in the thoracic spine in kyphotic posture. Exactly this posture is provided when a patient wears the physio-logic^® ^brace.

Techniques of delordosation have to be used for ILBP to support the instable segment by horizontalisation of the disc space at L5/S1 or, if needed at L4/5. Of course we have to recognize that this is a non-physiological posture, however pain reduction in those cases with symptomatic sagittal instability is the initial aim. Strengthening of the abdominal muscles and postural education regarding the optimum pelvic alignment to reduce sagittal shifting forces are the main focus in these conditions about 10% of the LBP population experience.

**Figure 5:**
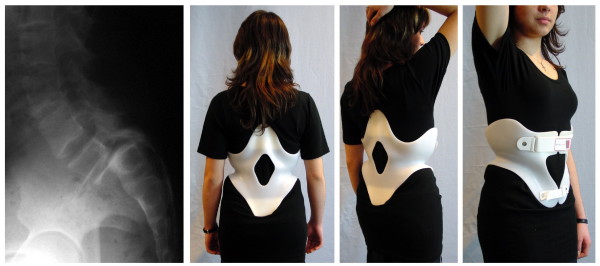
**spondylogic^® ^brace**. spondylogic^® ^brace in a patient with a scoliosis of less than 25° and a symptomatic spondylolisthesis. Immediate pain relief was dramatic and the symptoms of spinal stenosis in this 14-year old girl have been reduced drastically also. This patient still wears the brace for nearly 2 years and the reduction of the symptoms remains stable when she wears the brace during standing and walking. Mid- to long-term effects in general however are not yet reported upon for this brace.

The bracing strategy for this condition consequently leads to a reduction of lumbar lordosis with the help of a simple 3-point system (spondylogic^® ^brace) as can be seen on figure 5[23].

It seems that the artificial posture adolescents with symptomatic ILBP (eg. symptomatic spondylolisthesis) leads to a scoliosis in some cases perhaps due to the reduction of the sagittal profile, which is supposed to destabilise the segmental configuration and its function [22].

In individualised physical rehabilitation programs for low back pain, restoring function is the primary goal before specific bracing is offered, knowing that a loss of mobility correlates with an increase in pain intensity [24].

The effectiveness of a classification-based low back pain program is yet to be established [18], but this new and simple classification based on physical testing may help to establish specific low back pain programs in the future.

Further investigations are necessary with patients not suffering from scoliosis or other spinal deformities, in order to reveal whether this classification also can be used in general low back pain patients.

With the exception of 3 patients we have been able to distinguish between the two different types of 'unspecific low back pain' independent from the different kinds of spinal deformities. This finding seems to support the conclusion, as has been made in previous papers [13,15], that other factors other than the presence of spinal deformities contribute to chronic low back pain.

## Conclusion

Chronic unspecific low back pain is possible to clearly be classified physically in patients with spinal deformities. This functional classification is necessary to aid decision making as to which specific conservative approach (lordosating or delordosating physical therapy/braces) should be used.

Other factors than spinal deformities contribute to chronic low back pain.

## Consent

Written informed consent was obtained from the patients for publication of their pictures.

## Competing interests

HRW is a consultant for Koob-Scolitech.

MW does not declare any competing interests related to this paper.

## Authors' contributions

HRW: Patient acquisition, manuscript writing, statistics. MW: Patient acquisition, technical support.
